# Advances in Nano-Enhanced Thermal Functional Materials

**DOI:** 10.3390/nano16171095

**Published:** 2026-09-01

**Authors:** Peng Tao, Xiaoliang Zeng, Chao Chang

**Affiliations:** 1 State Key Laboratory of Metal Matrix Composites, School of Materials Science and Engineering, Shanghai Jiao Tong University, Shanghai 200240, China; 2 Shenzhen Institutes of Advanced Technology, Chinese Academy of Sciences, Shenzhen 518055, China; 3 Institute of Marine Engineering and Thermal Science, Marine Engineering College, Dalian Maritime University, Dalian 116026, China

Efficient thermal energy conversion, storage, and management are critical for global energy sustainability and the stable operation of modern electronic devices and industrial systems [1]. At the core of these applications are thermal functional materials, which dictate thermal transport, heat dissipation, and energy storage performance. Conventional thermal materials suffer from inherent performance limitations, such as low thermal conductivity and slow phase change kinetics [2]. The field of nanotechnology offers practical solutions for addressing these constraints via modifying microstructures and engineering nanoscale interfaces [3]. The integration of functional nanomaterials and nanostructures can enhance the thermophysical properties of base thermal materials and improve their operational performance. However, incorporating nanomaterials and nanostructures into thermal systems also introduces practical issues, including particle agglomeration, high interfacial thermal resistance, degradation during repeated thermal cycling, and processing costs [4]. Consequently, rational selection of nanomaterials and technology, appropriate synthetic routes, surface functionalization, and structural designs is essential for the effective deployment of nano-enhanced thermal functional materials in real-world applications [5].

This Special Issue, “*Advances in Nano-Enhanced Thermal Functional Materials*”, compiles eight featured contributions (six research articles and two reviews) spanning nano-enhanced thermal storage, high-conductivity nanocomposites, and additive-manufactured cooling devices, as well as specialized thermal sensing, low-temperature environmental catalysis, and plasmonic photothermal-managed flexible sensing platforms, offering valuable insights for the production of next-generation thermal energy technologies.

Nano-enhanced phase change materials (PCMs) and thermal storage constitute a major focus of this collection, addressing critical bottlenecks in heat storage capacity, thermal conductivity, and structural design to improve overall energy storage efficiency. In high-temperature quaternary molten nitrate salts, Zhu et al. demonstrated that incorporation of nano-SiO_2_/MgO particles with optimal compositions simultaneously enhanced specific heat capacity and thermal diffusivity [6]. Regarding structural optimization, Wang et al. utilized a combined experimental and numerical approach to reveal how micro-scale pore shapes in metal foams govern the melting and solidification behaviors of organic paraffin composite PCMs [7]. Complementing these primary studies, Tao et al. provided a comprehensive review of recent progress in enhancing the thermophysical properties of hydrated salts—another category of low-cost inorganic PCMs widely used at low-to-medium temperatures—through compounding with various carbon nanomaterials [8]. They systematically discuss representative fabrication strategies, selecting carbon nanofillers, and the improved thermal management performance of these carbon-enhanced PCM composites. Beyond conventional nano-scale composite optimization, exploring thermal storage mechanisms from a molecular dimension offers opportunities for fundamental breakthroughs. Recent progress demonstrates that utilizing strained molecular isomers can achieve high-density solar energy harvesting and controlled heat extraction under solvent-free conditions [9]. Concurrently, integrating photo-switching dopants into organic phase-change materials introduces an activation energy barrier that suppresses spontaneous heat loss, enabling optically triggered latent heat release below the crystallization point [10].

In addition, several contributions target advanced thermal management materials, interfaces, and cooling devices, focusing on passive cooling, packaging, and high-flux heat dissipation to mitigate localized heat concentration in high-power applications. Addressing the intrinsic thermal conductivity limits of carbon-fiber-reinforced plastic (CFRP) matrices, Bibinger and colleagues demonstrated that incorporating carbon nanotube buckypapers (8 wt.%) into CFRP laminates significantly enhances effective thermal conductivity and delays the onset of thermo-induced damage by up to 20% under high-flux thermal irradiation without compromising mechanical integrity [9]. Transitioning from composite materials to advanced cooling hardware, Ji et al. reported that leveraging 3D printing to fabricate flat-plate oscillating heat pipe finned radiators led to a 65.6% reduction in thermal resistance (down to 0.11 °C/W) relative to solid finned radiators while exhibiting excellent thermal performance across various inclination angles [10]. Through rational molecular-level design—such as utilizing rigid side-group dynamics [11], chain-orientation engineering reflected in ultra-drawn polyethylene [12], or simultaneous engineering of strong intramolecular covalent bonding and intermolecular interactions [13]—the intrinsic thermal conductivity of organic polymers can be enhanced without sacrificing structural homogeneity.

Finally, this collection highlights specialized thermal sensing, catalysis, and multifunctional nanostructures, showcasing the highly interdisciplinary applications of thermal functional materials, extending into diagnostics, environmental remediation, and flexible systems. Addressing the challenges of measuring rapid transient temperatures in micro/nanoparticle combustion, Li et al. developed a hybrid PMT/CMOS pyrometry system that accurately captures the temporal temperature evolution and interfacial flame characteristics of individual burning fuel particles (e.g., Al, Mg, B, and B_4_C) [14]. For environmental catalysis, Doroftei demonstrated that nanocrystalline iron manganite could act as a cost-effective alternative to noble-metal catalysts, achieving a 97% conversion rate for ethanol at 300 °C during thermal catalytic combustion [15]. This work highlights how nanoscale transition-metal oxides lower activation energy barriers for the thermal combustion of hazardous volatile organics (VOCs). Expanding the horizon of nanostructures into multifunctional flexible systems, Peng et al.’s review systematically evaluates stretchable SERS platforms, emphasizing how thermal management of plasmonic photothermal effects and elastomeric matrix stability enable strain-tunable hot-spot configurations for ultrasensitive, conformable molecular sensing [16]. Recent advances have further expanded this interdisciplinary frontier by coupling intrinsic thermal regimes with chemical and biological functions. Emerging work demonstrates that integrating photothermal or thermoelectric nanostructures into hydrogel and aerogel matrices can concurrently facilitate self-powered multiplexed biosensing for flexible diagnostics and accelerate interfacial mass transfer for solar-driven environmental remediation [17,18].

While the research featured here provides valuable examples, nano-enhanced thermal materials still present vast unexplored opportunities and pressing scientific challenges. Translating these materials from laboratory research into industrial implementation will require overcoming key manufacturing and modeling bottlenecks, including scalable, cost-effective nanomanufacturing and high-fidelity interfacial heat transport modeling. We express our sincere appreciation to all the authors and reviewers for their dedicated contributions. We hope this Special Issue serves as a constructive foundation that sparks further exploration and technological breakthroughs in regard to nano-enhanced thermal functional materials.
